# Supplementary material for: Reconfigurable metamaterial processing units that solve arbitrary linear calculus equations
Source: Nat Commun. 2024 Jul 24;15:6258. doi: 10.1038/s41467-024-50483-x (PMC11269748; doi:10.1038/s41467-024-50483-x)
Supplement: Supplementary file 1 — Supplementary Information [file 41467_2024_50483_MOESM1_ESM.pdf]

# Reconfigurable metamaterial processing units that solve arbitrary linear calculus equations

Fu. et al.

**This supplementary information contains the following sections:**

**Supplementary Note 1: Derivation of Eq. (1) in the main text.**

**Supplementary Note 2: Signal processing with calculus kernels directly.**

**Supplementary Note 3: Details of optimization algorithms.**

**Supplementary Note 4: Individual test results for calculus kernels.**

**Supplementary Note 5: Error analysis and robustness of MPU.**

**Supplementary Note 6: Discussion on processing time of MPU.**

**Supplementary Note 7: Discussion on power consumption of MPU**

**Supplementary Note 8: Discussion on solving integral equations.**

**Supplementary Note 9: Discussion on higher order expansion of equation solver.**

**Supplementary Note 10: FPGA control scheme and switching time.**

## Supplementary Note 1. Derivation of Eq. (1) in the main text.

We can use the method of signal flow graph to derive the S parameters of the system. As shown in Fig. S1(a), we have defined four key nodes. We assume that the operation performed by the core of the inverse design containing adjustable units is K and is non-reflective. In practical experiments, non-reflection is achieved through isolators. Consider that the direct parameter of the coupler is $s_1$, the coupling parameter is $s_2$, and the coupler is perfectly matched. The entire system can be represented by the signal flow diagram in the Fig. S1(b). Then, according to the theory of signal flow graph, the diagram can be simplified step by step, from Fig. S1(c) to Fig. S1(e). At last, the Eq. (1) in main text can be generated.

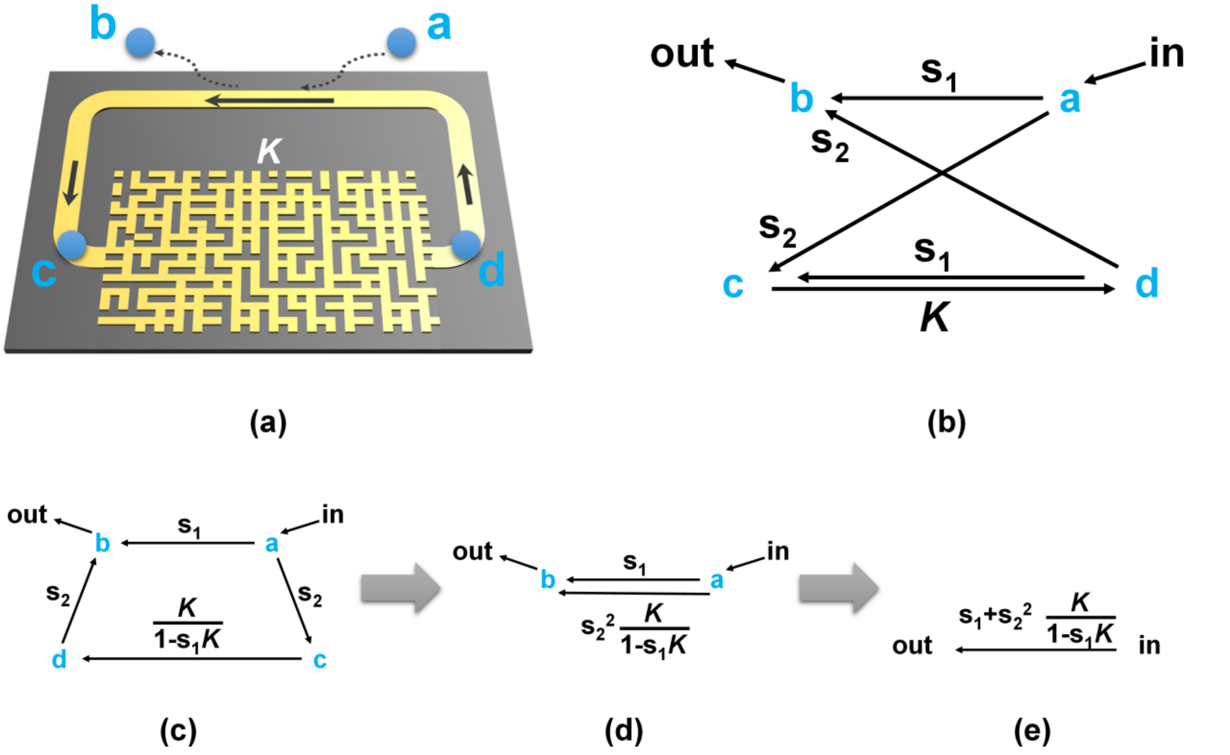


**Supplementary Figure 1 | Deducing the transmission function of the system through signal flow graph. (a)** Several key nodes in the concept map. Nodes a-b-c-d are four ports of the coupler. **(b)** Signal flow graph of the system. The coefficients of the coupler are defined as $s_1$ and $s_2$. **(c)(d)(e)** Simplify the signal flow graph step by step.

## Supplementary Note 2. Signal processing with calculus kernels directly.

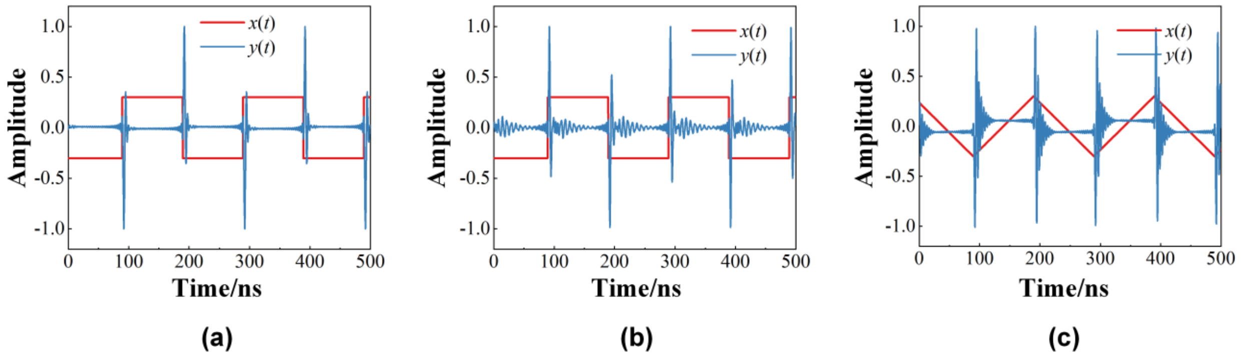


**Supplementary Figure 2 | Time-domain signal processing with differentiators. (a)** Directly process time-domain signals using the optimized first-order differentiator. A series of impulse response signals can be obtained by inputting a square wave signal with a period of 5MHz into the differentiator. This proves the effectiveness of the first-order differentiator. **(b)** Directly process time-domain signals using the optimized second-order differentiator. A series of double impulse response signals can be obtained by inputting a square wave signal with a period of 5MHz into the second-order differentiator. This proves the effectiveness of the second-order differentiator. **(c)** Directly process time-domain signals using the optimized third-order differentiator. A series of double impulse response signals can be obtained by inputting a sawtooth wave signal with a period of 5MHz into the third-order differentiator. This proves the effectiveness of the third-order differentiator.

## Supplementary Note 3: Details of optimization algorithms.

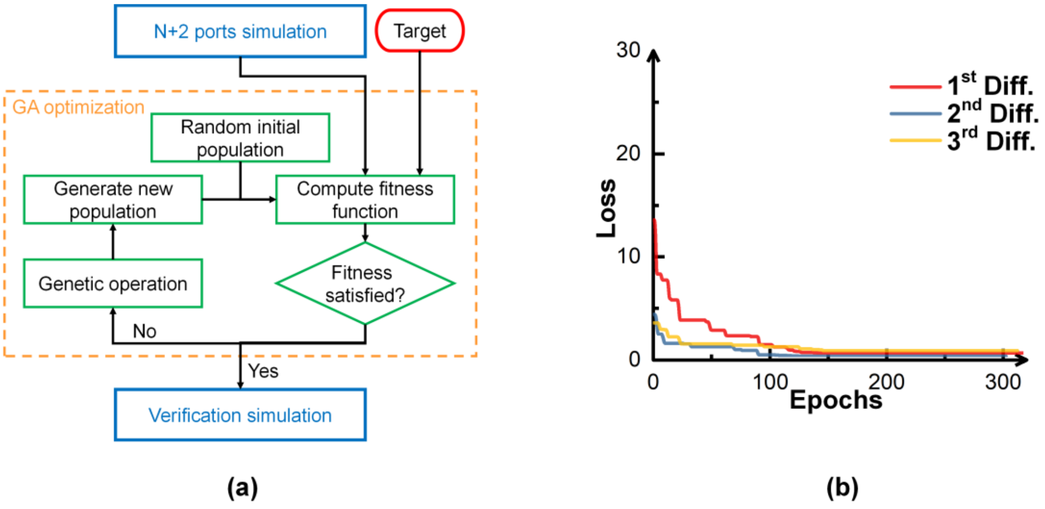


**Supplementary Figure 3 | Optimization process of differential kernels. (a)** The flowchart of the entire algorithm. The blue box represents the simulation in HFSS, the green box represents the optimization process in MATLAB, and the orange part uses classic genetic algorithms. **(b)** The change in fitness parameters during the optimization of first-, second- and third- order differentials. After 150 epochs, the loss tends to 0, achieving the optimization goal.

## Supplementary Note 4: Individual test results for calculus kernels.

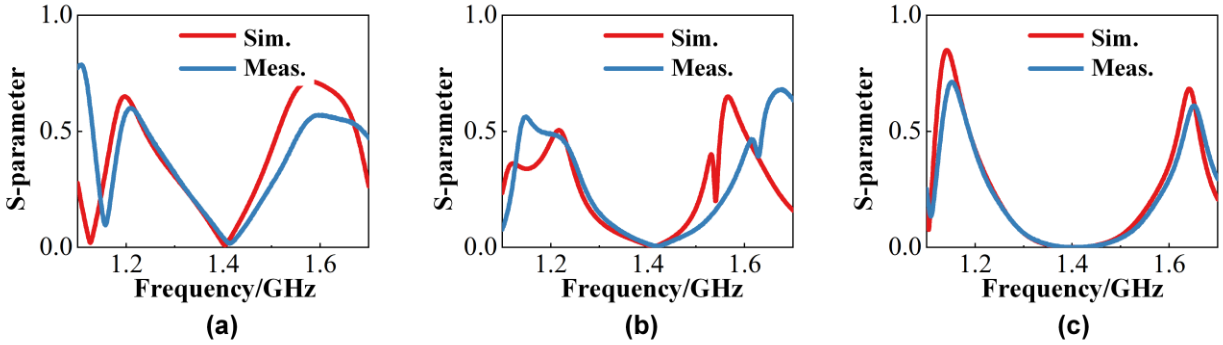


**Supplementary Figure 4 | Measurement results of each differential kernel.** **(a)** First-order differential kernel. **(b)** Second-order differential kernel. **(c)** Third-order differential kernel. There is a slight frequency offset and loss between the measured and simulated results, which may be caused by the dielectric constant error of the processed dielectric plate.

## Supplementary Note 5. Error analysis and robustness of MPU.

We have investigated the effect of several different factors on the accuracy. In order to quantitatively analyze the solution error, based on the content in the main text, we define the left and right sides of equations as $g(t)$ and $h(t) = s_1\boldsymbol{K}g(t)\text{-}s_1^2\boldsymbol{K}x(t)$ respectively. And we define the solving error as the following Eq. S1.

$$\frac{\int (g(t)-h(t))^2 dt}{\sqrt{\int g(t)^2 dt \int h(t)^2 dt}} \tag{S1}$$

Firstly, the effect on different input signals is investigated. Here, we use the same MPU states as in Figure 3(c) in the main text. Different time domain signals with different waveforms are used as input signals of the system. As shown in Supplementary Figure 5, a simple sinusoidal signal cos(ωt) + sin(3ωt), square wave signal, and triangular wave signal with the same frequency of 30MHz is used as examples. It is illuminated that quite accurate solution results for three different signals are generated, indicating the robustness of MPU with different input signals.

Secondly, the effect on the different frequencies of input signals is investigated. As shown in Supplementary Figure 6, three square waves with frequency of 30MHz, 60MHz, and 10MHz are input into the MPU. Although solutions to the equations are generated in all three cases, it can be seen that the error rate at 60MHz case is significantly increased. Besides, as shown in Supplementary Figure 7, when the frequency of the input signal increases, the error rate of the solution grows, which demonstrates that the error is caused by the bandwidth of the actual physical system.

At last, the effect on the different offsets of modulation frequency are investigated. Predictably, since the calculus kernels require the signal to be modulated at the exact zero frequency, the frequency offset has a very large impact on the solution accuracy. As shown in Supplementary Figure 8, as the modulation frequency deviates from the center frequency of the MPU system, the error rate increases rapidly.

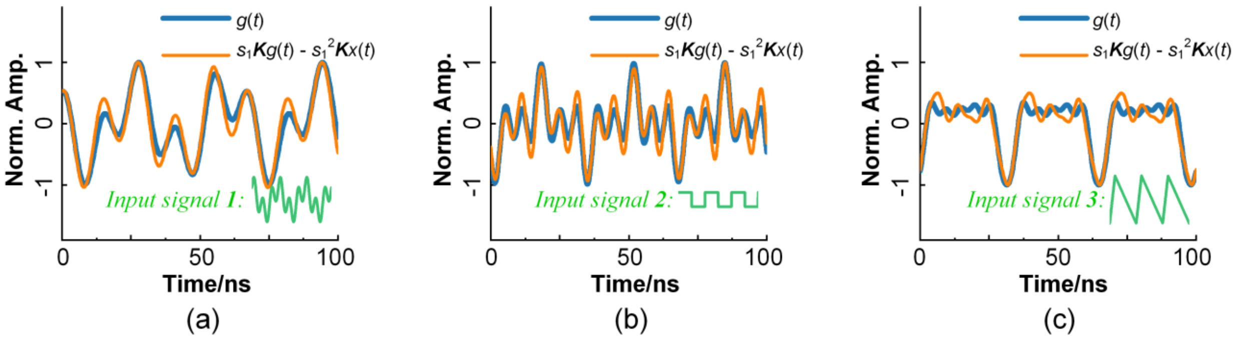


**Supplementary Figure 5 | Solutions of MPU with different input signals. (a)** The input signal is a superposition of simple sine waves. **(b)** The input signal is a square wave. **(c)** The input signal is a triangular wave.

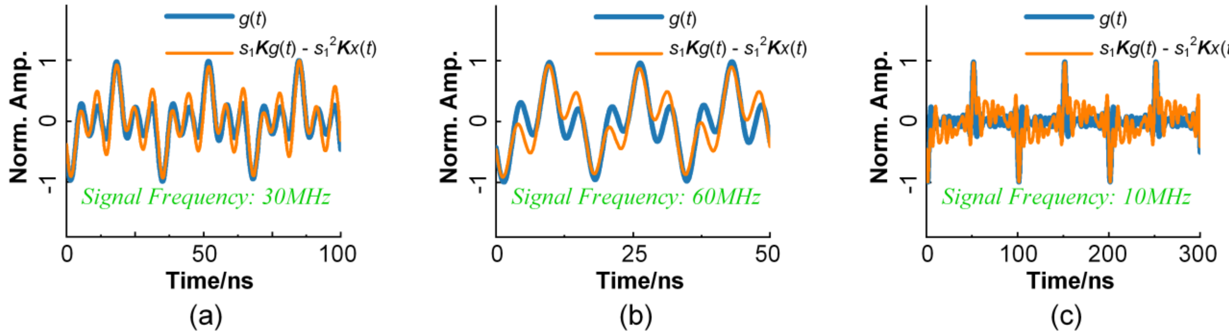


**Supplementary Figure 6 | Solutions of MPU with different frequency of input signals. (a)** The input signal is a square wave with frequency of 30MHz. **(b)** The input signal is a square wave with frequency of 60MHz. **(c)** The input signal is a square wave with frequency of 10MHz.

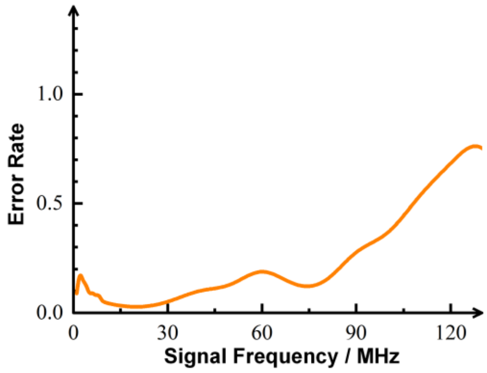


**Supplementary Figure 7 | Relationship between solving error rate and input signal frequency.** As the frequency of the input square wave signal varies, the error rate of the solution grows, which demonstrates that the error is caused by the bandwidth of the actual physical system.

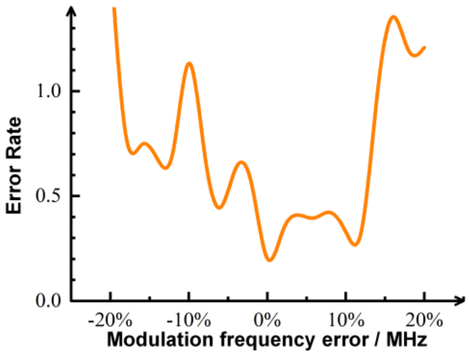


**Supplementary Figure 8 | Relationship between solving error rate and modulation frequency error.** As the modulation frequency of the input deviates from the center frequency point, the error rate of the solution grows quickly.

## Supplementary Note 6. Discussion on processing time of MPU.

To evaluate the processing speed of an equation solver, it is crucial to analyze the time required for the signal to achieve a stable state within the MPU. In order to conduct this analysis, we developed a simulation model using Advanced Design System® software, version 2015.01. The simulation explores the time-domain process across various states of the MPU. Differential calculus kernels, as illustrated in **Supplementary Figure 9** (a-c), were configured to exhibit first-order differentiation, both first and second-order differentiation, and all three orders of differentiation, respectively. In the simulation, an input signal with a center frequency of 1 GHz was utilized for generality. Results indicate that regardless of the MPU's state, the output signal representing the solution stabilizes after approximately 30 cycles. This analysis provides valuable insights into the processing speed of the equation solver and highlights the effectiveness of the MPU in achieving steady-state solutions within a reasonable timeframe.

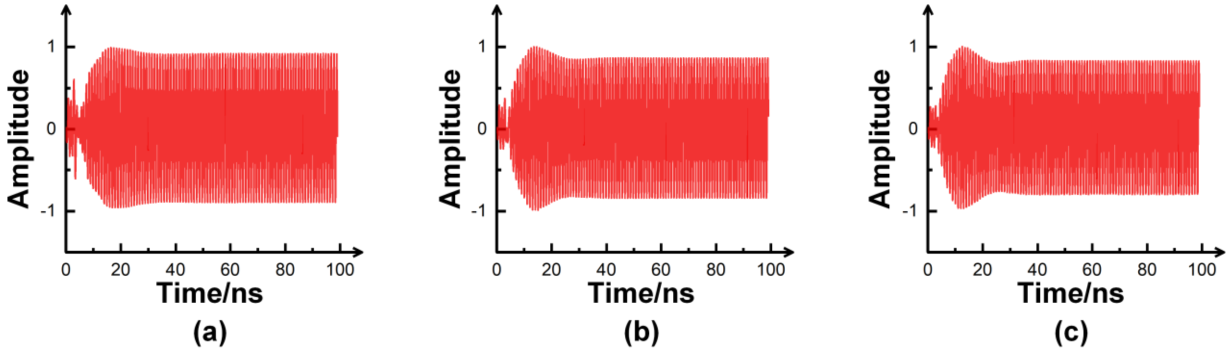


**Supplementary Figure 9 | The simulated output signals of three different kernel states.** **(a)** Only 1st order differentiation. **(b)** Both 1st and 2nd order differentiation. **(c)** All three different orders differentiations.

## Supplementary Note 7. Discussion on power consumption of MPU.

The power consumption of MPUs can be mainly composed of radio frequency (RF) signals, reconfigurable components, and field programmable gate array (FPGA) control devices. For RF signals, we generate a 1mW RF signal in the vector network analyzer as the input signal for experiment. For reconfigurable components, the QPA9126 chips used as amplifiers are with the input voltage of 5V and input current of 68 mA. Meanwhile, the PE44820 and PE43713 chips, used as programmable amplitude modulators and phase shifters, are with input currents of 150μA and 130μA, at an input voltage of 5V. Therefore, the total power consumption of reconfigurable components is 1024.2mW. For the FPGA control devices, the Arduino Mega 2560 Rev3 microcontroller used in the experiments is with input voltage of 5V and input current of 724.3mA. All in all, the total power consumption of the proposed MPU is about 11889.7mW. It should be noted that the vast majority of the power consumption comes from external control and configurable devices.

## Supplementary Note 8: Discussion on solving integral equations.

The "calculus equations" include differential equations, integral equations, and hybrid equations with both calculus operations. In the manuscript, we show the solution of general linear differential equations. There are two possible ways to solve equations containing integral operations. Firstly, as we derived in Section A of manuscript, ***K*** in the Eq. S2 can represent any calculus operation kernel.

$$h(t) = s_1 \boldsymbol{K} h(t) - s_1 x(t) \tag{S2}$$

When an integral kernel is constructed and integrated into the proposed architecture, the solution of the integral equation can be performed. However, an $n^{th}$ order integral operation requires a processing kernel with the following transmit function: $T(\omega) = [-j/(\omega - \omega_0)]^n$, which requires an infinite response at the center frequency point. This, on the one hand, makes the approximation of kernel inaccurate, and on the other hand, it may bring unstable self-excitation to the feeding back loop of proposed architecture. In addition to this, for linear integral equations, we can transform them into linear differential equations by taking derivatives on both sides and variable substitution. Since our solution results in a steady-state solution of the system, we can obtain the solution of the integral equation from the results of the corresponding differential equation and the input signal. As an example, following the RLC model shown in the manuscript, the current $i(t)$ in the circuit satisfies the following calculus equation:

$$L\frac{di(t)}{dt} + Ri(t) + \frac{1}{C}\int i(t)dt = u(t) \tag{S3}$$

Considering the calculus relationship between the current $i(t)$ and charge $q(t)$: $dq(t)/dt = i(t)$, we can obtain the current in the circuit by solving for the charge $q(t)$ that satisfies the differential equation.

$$L\frac{d^2q(t)}{dt^2} + R\frac{dq(t)}{dt} + \frac{1}{C}q(t) = u(t) \tag{S4}$$

## Supplementary Note 9: Discussion on higher order expansion of equation solver.

For arbitrary calculus kernels that typically have more orders, in addition to optimizing them using suitable pixel metamaterial structures, we can obtain them by cascading the already obtained lower order differentiators as well. As shown in **Supplementary Figure 10**, we provide an example of cascading to create higher order differential kernels. To eliminate reflected waves from mismatched individual differentiators, we need to add isolators or attenuators constructed from lossy media between the differentiators in the cascade. **Supplementary Figure 10** (b, c, d) show the results for 2$^{nd}$, 3$^{rd}$, and 8$^{th}$ order differentiators constructed from 1$^{st}$ order differential kernels and 5dB attenuators, respectively, and are in good agreement with expectations. Furthermore, with the planar structure of pixel metamaterials, it is also possible to construct higher-order kernels by stacking them in layers to reduce the overall size of the device.

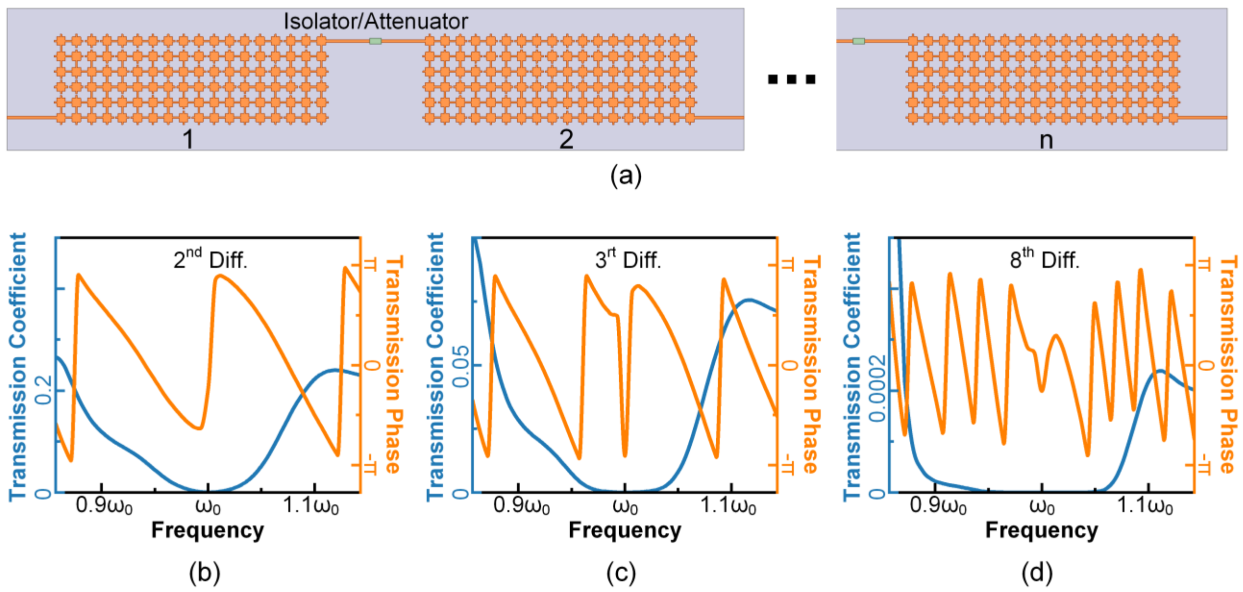


**Supplementary Figure 10 | Higher order expansion of equation solver. (a)** By cascading differential kernels and attenuators, higher order differential kernels can be realized. **(b)** The transmission coefficient and phase of the cascaded second order differential kernel. **(c)** The transmission coefficient and phase of the cascaded third order differential kernel. **(d)** The transmission coefficient and phase of the cascaded eighth order differential kernel.

## Supplementary Note 10: Discussion on FPGA control scheme and switching time.

The **Supplementary Figure 11** gives the principle of controlling the switching state of the system with FPGA. A commercial Arduino® Mega 2560 Rev3 microcontroller integrated with ATmega2560 processor is used as the controller. As shown in **Supplementary Figure 11 (a)**, this type of FPGA chips has multiple input and output ports. And as in **Figure 11 (c)**, the pins and feeding ports of the two chips used as amplifiers and phase shifters in the experiment are connected to the output ports of the FPGA.

As for the switching time, the delay of the MPU to switch among multiple states is mainly limited by several factors: the time required for the system to establish a steady-state solution, the switching time of the reconfigurable components, and the clock signal of the FPGAs. As we discuss in the **Supplementary Note 6**, the output signal representing the solution stabilizes after approximately 30 cycles, implying a settling time of about 21ns. In addition, the chips used for amplitude modulation and phase shifter have a settling time of 1600ns and 365ns respectively. Furthermore, the FPGA chip used in the experiments has a main frequency of 16MHz, implying a switching time of up to 62.5ns. Taken together, the switching time of the MPU is mainly limited by the settling time of the reconfigurable chips, which limits the whole MPU prototype to switch different solver states as fast as 25kHz. It should be noted that different reconfigurable components and materials are key to the switching speed of the MPU system.

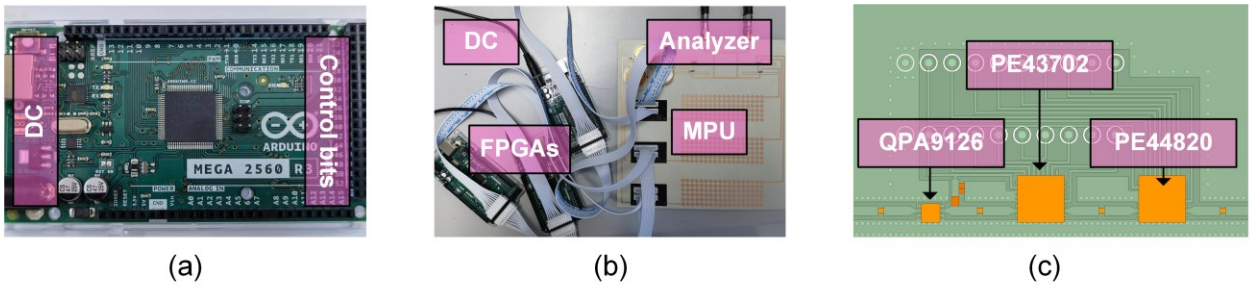


**Supplementary Figure 11 | FPGA control scheme. (a)** Commercial Arduino® Mega 2560 Rev3 microcontroller, integrated with ATmega2560 processor. **(b)** The FPGA is connected to the MPU via a row of wires. **(c)** Control circuit for MPU reconfigurable chips.
